# Hereditary Anatomical Risk Factors for Anterior Cruciate Ligament Injuries

**DOI:** 10.7759/cureus.55129

**Published:** 2024-02-28

**Authors:** Tetsuo Hagino, Satoshi Ochiai, Naoto Furuya, Tetsuhiro Hagino, Masanori Wako, Naofumi Taniguchi, Hirotaka Haro

**Affiliations:** 1 Department of Orthopedic Surgery, National Hospital Organization (NHO) Kofu National Hospital, Kofu, JPN; 2 Department of Orthopedic Surgery, University of Yamanashi, Chuo, JPN

**Keywords:** risk factors, intercondylar notch, family members, anatomical characteristics, acl injuries

## Abstract

Introduction: Genetic and anatomical factors have been reported as risk factors for anterior cruciate ligament (ACL) injuries. This study aimed to investigate anatomical characteristics in family members sustaining ACL injuries, compared with age- and sex-matched patients with simple meniscus injuries.

Materials and methods: Medical records of 1548 patients who underwent ACL reconstruction were reviewed. Cases of ACL injury occurring in first-degree relatives were selected. Forty-one patients from 20 families were included in the study (F-ACL group). Fifty patients with meniscus injuries were included as controls. Anatomical factors comprising posterior-inferior tibial slope (PITS), notch width index (NWI), notch angle (NA), and intercondylar notch roof inclination angle (RA) were compared between groups. The correlation of these anatomical factors between parent and child or siblings was also investigated.

Results: The 41 patients (20 families) consisted of 12 parent-child pairs and 29 siblings (13 pairs and one trio). Injuries occurred during playing the same sport in 11 families (55%). PITS was significantly steeper in the F-ACL group (9.9 vs. 7.8 degrees). NWI and NA were significantly smaller in the F-ACL group (0.262 vs. 0.278 and 50.5 vs. 58.8 degrees). RA was significantly greater in the F-ACL group (130 vs. 126.9 degrees). A positive correlation in NA (r = 0.677) and a weak correlation in NWI and RA were observed between family members.

Conclusions: Common anatomical risk factors of ACL injury exist within families, including intercondylar notch stenosis and steep posterior tibial slope. The findings suggest the potential for developing effective ACL injury prevention programs targeting these risk factors.

## Introduction

An anterior cruciate ligament (ACL) injury is a severe knee injury that can result in long-term morbidity, significantly impacting an individual's physical activity, quality of life, and ability to perform daily activities. It is a common injury affecting individuals engaged in a wide range of sports, particularly those involving jumping, cutting, and pivoting movements, such as basketball, soccer, football, and skiing. Elucidating risk factors of ACL injury is important for preventing ACL injury, improving treatment outcomes, and developing prevention programs aimed at eliminating risk factors. Many studies have been conducted on risk factors for the development of ACL injury. The Japanese Orthopaedic Association (JOA) Clinical Practice Guidelines on the Management of Anterior Cruciate Ligament Injury (3rd edition) indicate that sex (female), anatomic factors such as steeper posterior tibial slope, neuromuscular factors, genetic factors, race, and a family history of ACL injury are associated with risk of ACL injury [1].

While a family history of ACL injury has been reported as a potential risk factor [2-8], the existing evidence remains inconclusive. Conversely, the association of a steep posterior tibial slope with ACL injury risk has been documented [9,10]. A stenotic intercondylar notch can lead to impingement of the ACL on the lateral femoral condyle, exposing it to anterior shear force or tibial rotation, potentially resulting in rupture [11]. Several studies have highlighted the significance of a narrow intercondylar notch and a steep tibial slope as risk factors for ACL injury [12-20]. However, it's noteworthy that some studies have failed to establish a significant correlation between a narrow intercondylar notch and the risk of ACL tear [21,22]. Despite this wealth of literature, there remains a scarcity of research specifically exploring anatomical factors within families predisposed to ACL injury [4,23].

The primary purpose of this study was to identify anatomical characteristics of ACL injuries occurring in family members compared to patients with simple meniscus injuries, and the secondary purpose was to examine whether common anatomical risk factors are shared between parent and child or siblings of families with ACL injury.

## Materials and methods

This research was a retrospective longitudinal cohort study. Among 1,548 patients who underwent ACL reconstruction at our center between January 2006 and May 2022, we extracted cases of ACL injuries that occurred in family members up to first-degree relatives. A total of 41 patients (19 males and 22 females) from 20 families were included as subjects in the study. We investigated details of injuries occurring within these families and the causes of the injuries.

**Figure 1:**
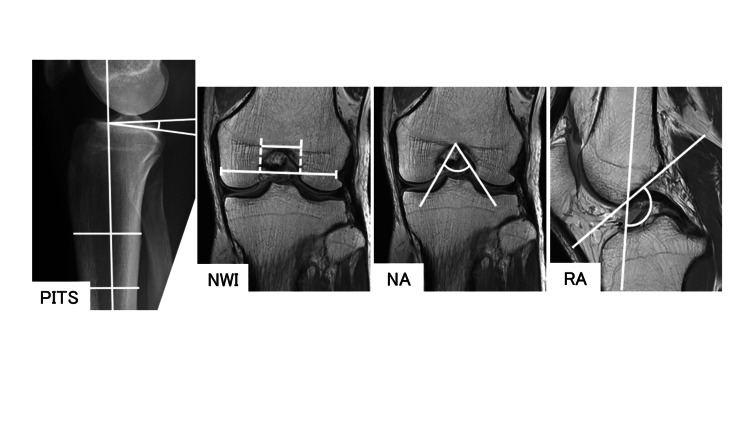
Anatomical factors The posterior-inferior tibial slope (PITS) angle is defined as 90°minus the angle made by the intersection of the line along the longitudinal axis of the tibia and the slope of the medial tibial plateau, as described by Kostogiannis et al. [24] The notch width index (NWI) is the ratio of the intercondylar notch width to the bicondylar width of the distal femur at the level of the popliteus groove in coronal view. The notch angle (NA) is measured at the level of the popliteus groove, tracing the opening of the intercondylar notch in coronal view. Intercondylar roof inclination angle (RA) is the obtuse angle formed by the intersection of a line over the Blumensaat line and a line parallel to the long axis of the knee in the midsagittal view. MWI, NA, and RA measurements are as described by Tuca et al. [18].

Additionally, anatomical factors, including posterior-inferior tibial slope (PITS), notch width index (NWI), notch angle (NA), and intercondylar notch roof inclination angle (RA), were measured (Figure 1).

PITS was measured on simple x-ray images following the method reported by Kostogiannis et al. [24]. NWI, NA, and RA were measured on MR images according to the methods of Tuca et al. [18]. These measurements were compared between the group of ACL injuries occurring in families and a control group comprising 50 age- and sex-matched patients who underwent surgery for simple meniscal injury without other concurrent injuries such as ligament rupture, fracture, or severe cartilage injury after 2006. Furthermore, a single correlation coefficient between parent and child or between siblings was obtained for each anatomical factor.

For statistical analysis, data between two groups were conducted using the χ2 test, or two-sample t-test. Additionally, we examined the correlation in anatomical factors between family members using univariate correlation analysis and calculated the correlation coefficients. StatFlex ver. 7 software was used for all statistical analyses. A p-value less than 0.05 was considered statistically significant.

## Results

Forty-one patients from 20 families were included in the study. Twelve patients from six families were parents and children, comprising three pairs of mother and daughter, two pairs of mother and son, and one pair of father and son. Twenty-nine patients of 14 families were siblings, comprising five pairs of brother and younger sister, three pairs of brothers, three pairs of sisters, two pairs of sister and younger brother, and one trio of brother and younger sister and younger brother. Regarding the causes of injury in the 41 patients, 36 patients had sports-related injuries caused by basketball in 10 patients, volleyball in 10 patients, judo in six patients, soccer in three patients, badminton in two patients, rugby in two patients, karate in two patients, and handball in one patient; while four patients had injuries caused by falling, and one patient by riding a bicycle. Patients in 11 of the 20 families (55%) were injured during the same sport: volleyball in four families, basketball in three families, judo in two families, karate in one family, and badminton in one family. Twenty-nine of the 41 patients had non-contact injuries.

Comparing the group with ACL injuries occurring within the family (F-ACL group, n = 41) and the group with simple meniscus injury (control group, n = 50), the mean age was comparable in the F-ACL group and control group (22.9 years vs. 24.2 years), and there were no differences in sex ratio and side of injury side between the two groups (Table 1).

**Table 1:** Demographic comparison of familial anterior cruciate ligament (F-ACL) injury and control groups. Data are presented as mean standard deviation or number of patients.

Demographics		F-ACL injury group (n=42)	Control group (n=50)	p-value
Age, years, mean±SD (range)		22.9±9.9（15-53）	24.2±9.2（15-50）	0.513
Sex				
	Male	19	20	0.543
	Female	22	30	
Affected knee				
	Left	23	24	0.442
	Right	18	26	

 

**Figure 2:**
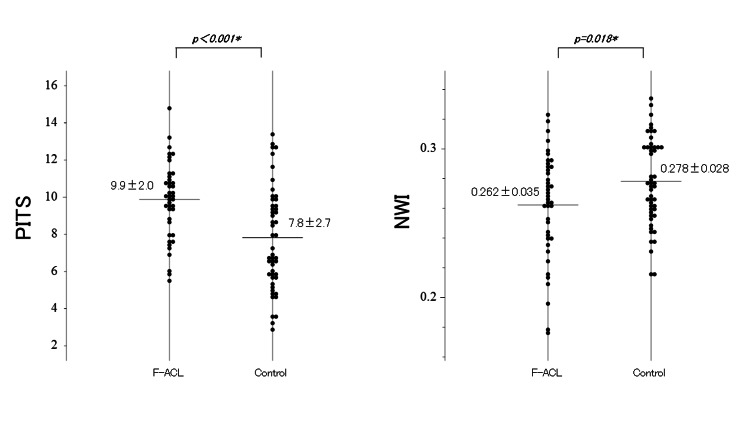
Comparing the anatomical factors (PITS, NWI) Comparisons between F-ACL group (n = 41) and control group (n = 50) in posterior-inferior tibial slope (PIST) and notch width index (NWI). Horizontal bar indicates mean value. Data expressed in mean ± standard deviation is shown next to each dot plot. *p＜0.05, statistically significant difference.

In comparing the anatomical factors, PITS was 9.9±2.0 in the F-ACL group and 7.8±2.7 in the control group and was significantly steeper in the F-ACL group (p < 0.001). NWI was 0.262±0.035 in the F-ACL group and 0.278±0.028 in the control group, and was significantly smaller in the F-ACL group (p＝0.018) (Figure 2).

**Figure 3:**
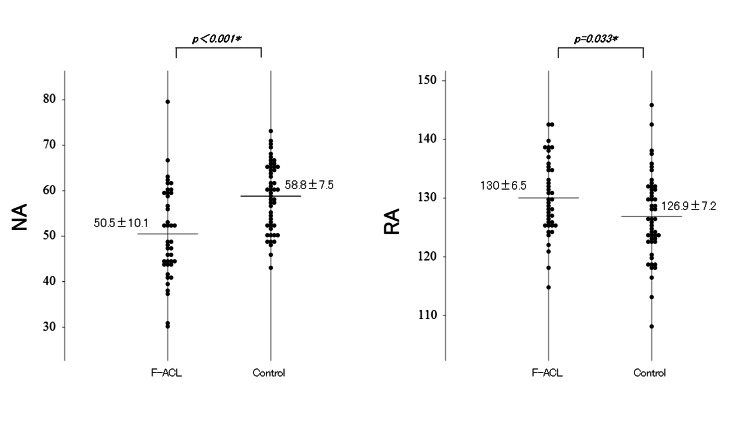
Comparing the anatomical factors (NA, RA) Comparisons between F-ACL group (n = 41) and control group (n = 50) in notch angle (NA), and roof inclination angle (RA). Horizontal bar indicates mean value. Data expressed in mean ± standard deviation is shown next to each dot plot. *p＜0.05, statistically significant difference.

NA was also significantly smaller in the F-ACL group than in the control group (p < 0.001). Furthermore, RA was 130±6.5 degrees in the F-ACL group and 126.9±7.2 degrees in the control group and was significantly steeper in the F-ACL group (p ＝0.033) (Figure 3).

**Figure 4:**
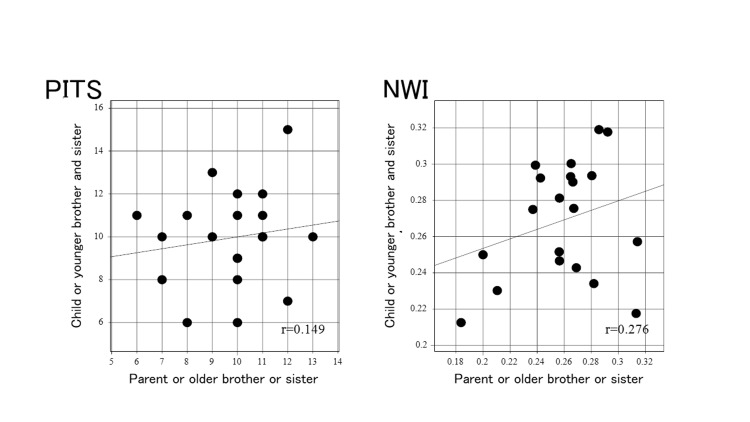
Correlation between family members for each anatomical factor (PTIS, NWI) Posterior-inferior tibial slope (PITS) or notch width index (NWI) was plotted with values for the parent or older brother or older sister on the horizontal axis, and the values for the child or younger sister or younger brother on the vertical axis. r = correlation coefficient.

For the analysis of the correlation of the anatomical factors between family members, no correlation between family members was detected for PITS. On the other hand, a weak correlation between family members was found for NWI (Figure 4).

**Figure 5:**
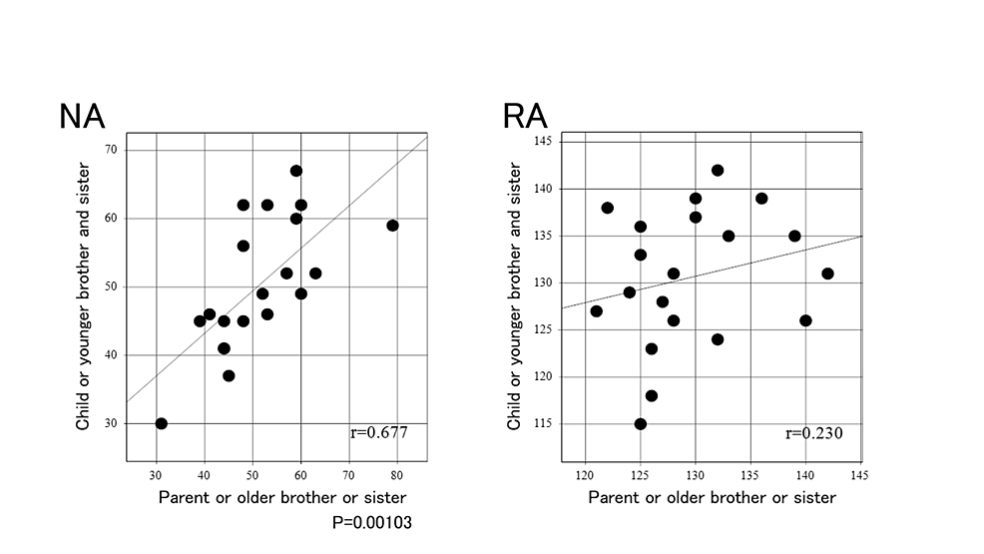
Correlation between family members for each anatomical factor (NA, RA) Notch angle (NA), or intercondylar roof inclination angle (RA) was plotted with values for the parent or older brother or older sister on the horizontal axis, and the values for the child or younger sister or younger brother on the vertical axis. r = correlation coefficient.

Furthermore, a positive correlation between family members was observed for NA (r = 0.677, p=0.00103), and a weak correlation for RA (Figure 5).

## Discussion

In a study on the family history of ACL injury, Goshima et al. [4] reported a high probability of familial predisposition to many of the identified risk factors for ACL injury. They also suggested that patients with a family history of ACL injury might be at high risk for initial and repeat ACL injuries. In a study of elite alpine skiers, Westin et al. [8] found that the skiers were more likely to have ACL injuries if they had a parent who had a history of ACL injury. Furthermore, Bram et al. [2] reported that children with a family history of ACL injury had a higher incidence of ACL tear. Indeed, a recent systematic review also reported that an individual with a family history of ACL injury had 2.5 times greater odds of sustaining an ACL injury than an individual without a family history [25]. Using a twin study approach, Magnusson et al. found that the overall heritability of ACL tear was high at 69%, and the familial risk was higher in identical twin pairs than in fraternal twin pairs [26]. This observed pattern of frequent ACL injuries within families, as indicated by the collective results of the cited studies, implies a notable tendency for familial clustering. Recognizing this familial predisposition could potentially empower clinicians to offer more targeted and effective counseling to athletes at an elevated risk of ACL tears. In addition to genetic factors, environmental factors such as sports participation and physical activity may also contribute to the occurrence of ACL injury within the family. In the present study, two or more family members were injured while participating in the same sport in over one-half of the families studied, suggesting that sport-specific factors may play a role in the occurrence of ACL injuries within these families.

Regarding anatomical factors, many reports have indicated that a narrow intercondylar notch and a steep posterior tibial slope are risk factors for ACL injury [9-12,14-17,20]. Tuca et al. [18] reported that a smaller, narrower, and steeper intercondylar notch may increase of risk of ACL reconstruction failure. A meta-analysis conducted by Zeng et al. rigorously examined the relationship between intercondylar notch dimensions and the risk of ACL injury. Analyzing 16 studies involving 4,291 participants, they found significant associations, demonstrating that narrow intercondylar notches, as indicated by lower intercondylar NWI and intercondylar notch width (NW), are linked to an elevated risk of ACL injury [27]. These results emphasize the critical role of specific intercondylar notch characteristics in ACL injury risk, providing valuable insights for our understanding of anatomical factors in this context.

There are few reports on anatomical factors in patients with ACL injuries occurring within the family. Keays et al. investigated the intercondylar NWI in siblings with ACL injuries compared with siblings with no injury and reported that the index was significantly smaller in the sibling group with injury [23]. Goshima et al. compared patients with a family history of ACL injury and those without a family history and found that the tibial slope was significantly steeper in patients with a family history of ACL injury [4]. Our results showed a significant correlation in NA and a weak correlation in NWI and RA between family members with ACL injuries, suggesting that family members with ACL injuries share similar morphology of the intercondylar notch. 

The positive correlation observed in NA within families suggests that there may be a genetic component to this anatomical factor. The intercondylar NA is determined by the size and shape of the intercondylar notch, which is formed by the bony ridges of the femoral condyles [18]. It may increase the strain on the ACL during dynamic movements and decrease the ability of the ACL to resist rotational forces.

This study demonstrated that patients with a family history of ACL injury might be at high risk for ACL injuries, with a genetic predisposition likely playing a role in the injury. The anatomical factors examined in this study; namely, PITS, NWI, NA, and RA, were found to be associated with ACL injury. Specifically, steeper PITS, smaller NWI and NA, and higher RA were associated with ACL injuries. The findings suggest that these anatomical factors may be useful in identifying individuals at risk of ACL injury and could be targeted in preventive measures. In this respect, Keays et al. [23] recommended radiological screening for siblings of ACL-injured athletes with narrow notches, and providing counseling, if necessary, regarding preventive training as a measure to reduce ACL injuries. The weak correlation between family members in some of the anatomical factors observed in this study suggests that other variables such as environmental and lifestyle factors may also contribute to the risk of ACL injury.

While this study provides valuable insights into familial predisposition to ACL injuries and associated anatomical factors, several limitations should be acknowledged. First, the retrospective design of the study hinders the establishment of causality, preventing definitive conclusions regarding whether the observed anatomical factors directly cause ACL injuries. Second, the relatively small sample size and the exclusive recruitment from a single institution may constrain the generalizability of the findings to broader populations. Additionally, the study primarily focused on genetic and anatomical factors, leaving a gap in the exploration of other potential contributors, such as environmental and lifestyle factors. To comprehensively understand the multifactorial nature of ACL injuries and enhance the development of effective preventive measures, further research with larger, diverse cohorts is warranted.

## Conclusions

Common anatomical risk factors of ACL injury were identified within families, including intercondylar notch stenosis and steep posterior tibial slope. These findings suggest the potential for developing effective ACL injury prevention programs targeting these risk factors.
